# Two cases of fungal cyst infection in ADPKD: is this really a rare complication?

**DOI:** 10.1186/s12879-019-4444-y

**Published:** 2019-10-29

**Authors:** Laura Onuchic, Victor Augusto Hamamoto Sato, Precil Diego Miranda de Menezes Neves, Bruno Eduardo Pedroso Balbo, Antônio Abel Portela-Neto, Fernanda Trani Ferreira, Elieser Hitoshi Watanabe, Andreia Watanabe, Maria Cláudia Stockler de Almeida, Leonardo de Abreu Testagrossa, Pedro Renato Chocair, Luiz Fernando Onuchic

**Affiliations:** 1 Department of Medicine, Division of Nephrology, University of São Paulo School of Medicine, Avenida Doutor Arnaldo, 455 – Sala 4304, São Paulo, SP 01246-903 Brazil; 2 Nephrology and Internal Medicine Service, Oswaldo Cruz German Hospital, São Paulo, Brazil; 3 Division of Infectious Diseases, University of São Paulo School of Medicine, São Paulo, Brazil; 4 Division of Pathology, University of São Paulo School of Medicine, São Paulo, Brazil

**Keywords:** Autosomal dominant polycystic kidney disease, Cyst infection, Fungal infection, *Candida albicans*, Antifungal treatment

## Abstract

**Background:**

Cyst infection is a prevalent complication in autosomal dominant polycystic kidney disease (ADPKD) patients, however therapeutic and diagnostic approaches towards this condition remain unclear. The confirmation of a likely episode of cyst infection by isolating the pathogenic microorganism in a clinical scenario is possible only in the minority of cases. The available antimicrobial treatment guidelines, therefore, might not be appropriate to some patients.

**Case presentation:**

We describe two unique cases of kidney cyst infection by *Candida albicans,* a condition that has not been previously described in literature. Both cases presented clear risk factors for *Candida* spp. infection. However, since there was no initial indication of cyst aspiration and culture, antifungal therapy was not immediately started and empirical treatment was initiated as recommended by the current guidelines. Antifungal treatment was instituted in both cases along the clinical course, according to their specificities.

**Conclusion:**

Our report highlights the possibility of *Candida* spp. cyst infection. Failure of clinical improvement with antibiotics should raise the suspicion of a fungal infection. Identification of infected cysts should be pursued in such cases, particularly with PET-CT, and when technically possible followed by cyst aspiration and culture to guide treatment. Risk factors for this condition, such as *Candida* spp. colonization, previous antimicrobial therapy, hemodialysis, necrotizing pancreatitis, gastrointestinal/hepatobiliary surgical procedure, central venous catheter, total parenteral nutrition, diabetes mellitus and immunodeficiency (neutropenia < 500 neutrophils/mL, hematologic malignancy, chemotherapy, immunosuppressant drugs), should be also considered accepted criteria for empirical antifungal therapy.

## Background

Autosomal dominant polycystic kidney disease (ADPKD) is the most common monogenic kidney disorder, affecting approximately 1 in 1000 individuals [1–3]. Urinary tract infections (UTI) are among the most prevalent complications in this disease, occurring in 30–50% of the patients throughout their lifetime [4, 5]. Renal cyst infection (CI) is of particular interest in this scenario, since it is associated with significant morbidity and mortality [6, 7]. Gram-negative bacteria are the typical causing agents in these episodes, most often related to the ascending urinary tract route [8, 9]. Interestingly, the urine sediment may be bland and urine culture negative in CI, because some cysts are not contiguous to the collecting system [1, 10]. While the diagnosis of this condition may be difficult, in recent years positron emission tomography/computed tomography (PET-CT) has become the most sensitive imaging technique to establish it [1, 3, 8, 11].

Although less usually, alternative etiologic agents and infectious routes have been also associated with renal CI in several reports [10, 12]. Fungal CI, however, is recognized as a rare event in ADPKD, with very limited prior description [13, 14]. Notably, in this report we present two cases of fungal CI with distinct outcomes, bringing attention to *Candida albicans* as a potential causative agent in CI and to the possibility that this complication be more common than currently assumed.

## Case presentation

### Case 1

A 34-year-old female with ADPKD was referred for persistent fever and malaise for the past 33 days. She reported symptoms and signs consistent with vaginal candidiasis 2 months prior to admission followed by right kidney obstruction secondary to ureteral calculus, which led to double-J stent placement. Two days after the procedure she developed fever of 101 °F and diffuse right abdominal pain, still sustained at admission. No urinary or gastrointestinal symptoms were reported. Urine and blood cultures were negative; white blood cell count and C-reactive protein (CRP), however, were elevated (14,650/mm^3^ and 389 mg/L, respectively). Initial computed tomography (CT) was inconclusive for CI. The patient was placed on broad-spectrum antibiotic regimens for the following 2 months, including intravenous (IV) ciprofloxacin 500 mg bid for 5 days, IV cephepime 1 g bid for 6 days, IV imipenem 500 mg bid plus metronidazole 500 mg tid for 20 days, and IV meropenem 1 g tid plus vancomycin 1 g bid plus fluconazole 200 mg sid for 4 days. Despite this treatment, she displayed no clinical improvement and developed acute kidney injury likely secondary to unresolved infection and potentially to vancomycin nephrotoxicity, with a rise in serum creatinine from 0.38 to 2.3 mg/dL. The laboratory tests are summarized in Table 1.

**Table 1:** Summary of the main patients’ laboratory tests

Laboratory Tests	Case 1	Case 2
Serum Creatinine at admission	2.3 mg/dL	2.3 mg/dL
White Blood Cell Count at admission	14,650/mm^3^	9040/mm^3^
Reactive C-Protein at admission	389 mg/L	102.9 mg/L
Urine Leucocytes at admission	> 100/field	49,000/mL
Urine Red Blood Cells at admission	> 100/field	5000/mL
Blood Culture	Negative	Negative
Urine Culture	*Candida albicans*	Negative
Cyst Aspiration Culture	*Candida albicans*	*Candida albicans*

**Fig. 1:**
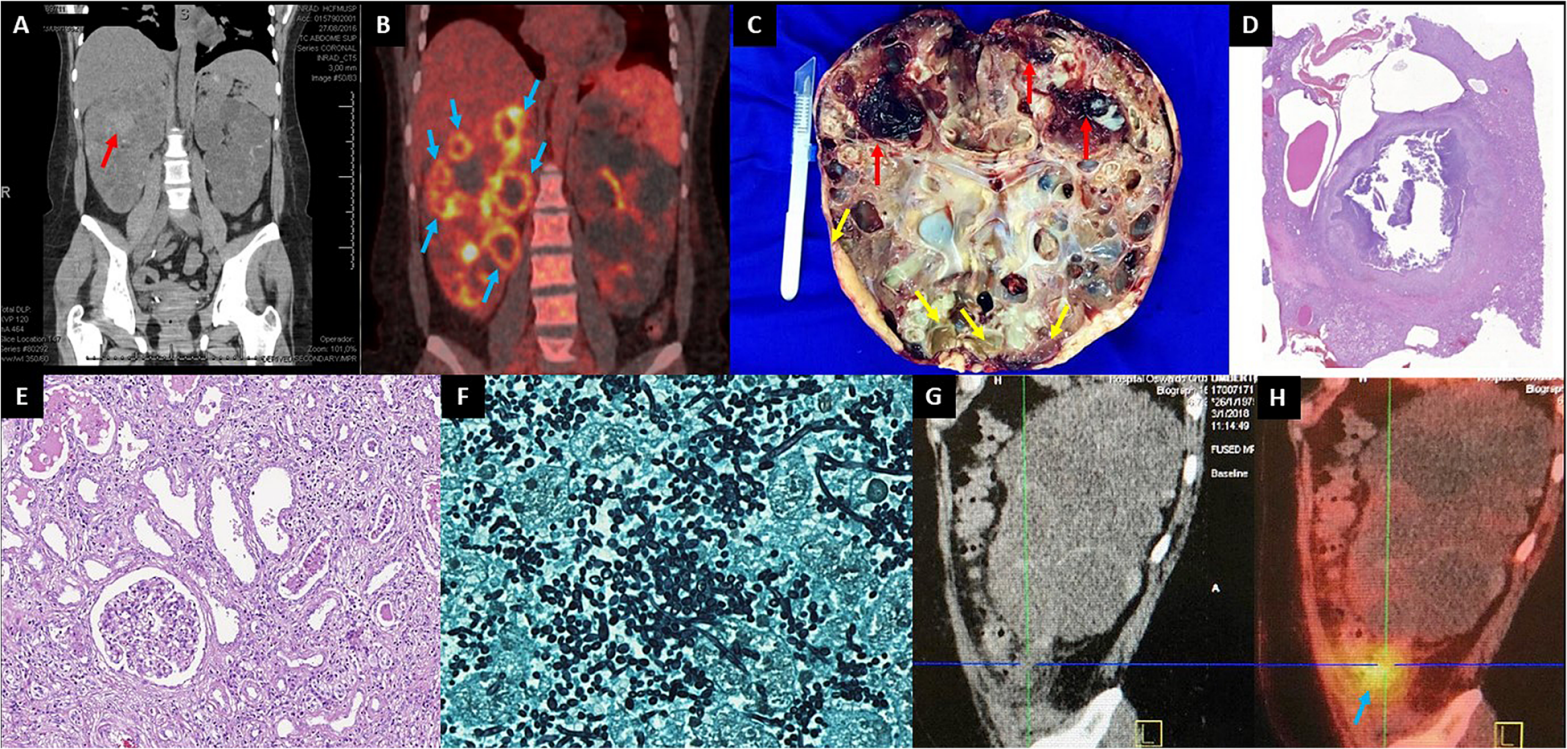
A prior study [11] revealed that ultrasound, CT and magnetic resonance imaging (MRI) failed to detect the infected cysts in 94, 82 and 60% of the cases, respectively. PET-CT, on the other hand, showed sensitivity of 100% on the basis of FDG uptake by inflammatory cells. Such data are in agreement with a previous report of ours [10]. Both of our cases support such a diagnostic capacity of PET-CT. **a** Non-contrasted computed tomography (CT) scan on coronal view shows no evidence of cyst infection. Some cysts present higher density, a finding suggestive of recent hemorrhage (*red arrow*); **b** Positron emission tomography–computed tomography imaging: coronal section analysis reveals increased cyst-lining 18-FDG uptake activity (*blue arrows*), a very suggestive finding of cyst infection; **c** Gross appearance of the right kidney after nephrectomy. This enlarged kidney presented multiple medium and large-sized cysts filled with pus (*yellow arrows*) or blood (*red arrows*); Histologic analysis of kidney section evidenced **d** an area of inflammatory cavitation, centered on urinary tract and adjacent to some cysts on the left superior field (hematoxylin-eosin, × 4 obj.); **e** Kidney parenchyma with neutrophilic interstitial infiltration, acute tubular damage and tubular neutrophilic casts (hematoxylin-eosin, × 20 obj.); **f** Numerous septate hyphae and yeast microorganisms with morphological features of *Candida sp.,* positively stained by Grocott’s methenamine silver (× 100 obj.); **g** Sagital view of enlarged kidneys with multiple cysts containing homogeneous liquid or heterogeneous dense hyperproteic material; **h** Imaging assessment reveals perirenal fascia thickening and high FDG uptake in an exophytic cyst (*blue arrow*), yielding the diagnosis of renal cyst infection

Once transferred to our center, the patient was submitted to a positron emission tomography-computed tomography (PET-CT) scan (Fig. 1a) which revealed high F-18 Fluor-deoxi-glucose (FDG) uptake in multiple right kidney cysts (Fig. 1b). Ultrasound-guided percutaneous drainage of the dominant suspected cyst followed by direct Grocott’s methenamine silver staining of the cyst content led to the diagnosis of fungal elements consistent with *Candida* spp., followed by isolation of *Candida albicans* from culture of the collected material. She was then immediately restarted on IV fluconazole 200 mg sid**.** During a 10-day period of this therapy, however, the patient maintained fever and malaise, associated with weight loss, progressive decline in renal function and very high levels of serum C-reactive protein (153–310 mg/L, for a normal reference range below 5 mg/L), with no tendency towards improvement. Given the refractoriness to this treatment, she was submitted to right nephrectomy (Fig. 1c, an estimated 1380-mL kidney), which led to renal failure and initiation of hemodialysis. Histopathology analysis confirmed this finding, showing cystic and pericystic hypha and pseudohypha invasion (Fig. 1d-f). At that moment she presented an unexpected tonic-clonic seizure not related to a significant electrolytic or hemodynamic disbalance but instead to a non-aneurysmatic subarachnoid hemorrhage documented by CT, which prompted her transfer to the intensive care unit.

Prolonged hospitalization led to urinary infection by carbapenemase-resistant *Klebsiella pneumoniae* followed by septic shock. An over two-week therapy with broad-spectrum antibiotics (IV tigecycline 50 mg bid plus ertapenem 1 g sid plus meropenem 1 g bid plus PO phosphomycin 3 g sid each 3 days) led to no clinical improvement and a new CT revealed multiple left kidney cysts with enhanced wall thickening. The patient eventually required high doses of vasopressors. In this context, left nephrectomy was performed (1093-mL kidney), resulting in clinical improvement. When recovering from surgery, however, she presented left-lower-limb deep venous thrombosis, requiring systemic anticoagulation. Critical falls in hemoglobin levels during several attempts of this intervention, in turn, made necessary the placement of a cava vein filter. After a debilitating eight-month period, she was discharged to her local medical facility for chronic hemodialysis still presenting significant frailty. Two months later, however, she died following a diagnosis of acute pancreatitis.

### Case 2

A 34-year-old male with ADPKD was admitted due to abdominal pain for the past 2 days, predominantly in the right flank and associated with temperature reaching up to 99.6 °F. His medical history included the diagnosis of arterial hypertension and chronic kidney disease stage IIIB (CKD-EPI of 37 mL/min/1.73m^2^) as well as an episode of intestinal perforation followed by enterectomy in 2013. The patient had been hospitalized twice during the previous 2 months due to UTIs associated with CI and renal function decline. He clinically improved in both occasions following treatment with IV linezolid 600 mg bid plus meropenem 1 g tid for 6 weeks, being discharged to complete antibiotic treatment on a home-care basis.

His serum creatinine was 2.3 mg/dL on admission, white blood cell count was 9040/mm^3^ and CRP 102.9 mg/L, while urinalysis showed 49,000 leucocytes/mL and 5000 erythrocytes/mL (Table 1). CT scan revealed obstructive acute abdomen secondary to bridles, pneumoperitoneum and gas bubbles in fat tissue adjacent to the transverse colon and hepatic angle. He was therefore submitted to laparotomy, bridle lysis and cavity washing. The surgical intervention was successful however CRP levels remained high, supporting the performance of PET-CT. Enlarged kidneys with multiple cysts containing homogeneous liquid or heterogeneous dense hyperproteic material were detected (Fig. 1g). This imaging assessment also revealed perirenal fascia thickening and high FDG uptake in a left 6.9 × 5.5 × 4.1-cm exophytic cyst, establishing the diagnosis of renal CI (Fig. 1h). CT-guided percutaneous cyst drainage allowed culture of its content, leading to isolation and diagnosis of *Candida albicans* CI. Blood cultures were negative. Following treatment with fluconazole for 6 weeks, the patient evolved with full clinical and laboratorial recovery, requiring no additional invasive procedures.

## Discussion and conclusion

Renal and/or hepatic CI is a common complication in ADPKD, with an incidence of approximately 0.01 episode/patient/year [11, 15]. Several of its aspects, however, remain not well defined, including diagnostic criteria and methods as well as treatment options and regimens [6, 9, 12]. Previous studies propose that a definite diagnosis of CI should be based on cyst aspirate showing neutrophil debris and/or a pathogenic microorganism [6, 16]. With minor variations, a common algorithm defines as likely CI the presence of fever (temperature > 38.5 °C for > 3 days); abdominal pain (particularly a palpable area of renal or liver tenderness); CRP > 50 mg/L, and absence of recent, significant intracystic bleeding or other causes of fever [1, 3, 11, 16, 17]. Since positive cyst cultures are usually not available, likely CI is far more prevalent than definite CI in the clinical scenario. Some reports detected a high prevalence of cyst infection among patients with advanced stages of chronic kidney disease. Indeed, the first case series using PET-CT to investigate cyst infection in ADPKD reported that 12 out 36 (33,3%) patients were on dialysis support [11], a finding in accordance with our previous report that identified 6 out 24 patients in this condition [10]. Of note, neither of the two currently reported cases developed such a complication while on dialysis.

Antibiotic treatment algorithms targeting CI are not robustly defined, since the infectious agent is isolated in a minority of cases. Jouret et al. [8] confirmed the pathogenic bacteria with cyst fluid cultures in only 3 of the 27 analyzed likely CI episodes while Sallee et al. [11] reported five positive cyst cultures among 41 evaluated CI events. In this scenario, different antibiotic approaches have been proposed and used, however none of them includes antifungal drugs as part of initial and second-line regimens. Based on their appropriate concentration within the cyst and antibiotic spectrum against gram-negative bacteria, currently proposed schemes classically include fluoroquinolones, particularly ciprofloxacin or levofloxacin [1, 3, 6, 7, 9]. Failure to improve with antibiotics, however, should raise the suspicion of a fungal infection. There is no consensus, nevertheless, whether routine empirical antifungal therapy should be administered in non-neutropenic patients, even if they are critically ill, since most studies in this specific group have not shown a survival benefit [18]. In our cases, however, both patients had risk factors accepted as criteria for empirical antifungal therapy. Invasive candidiasis is a critical condition associated with mortality in the same level as septic shock (40–60% mortality-rate). In this context, the presence of well-defined risk factors for this condition should always be evaluated by the designated clinician, especially in an inpatient setting. Identifying risk factors may lead to a quick and accurate diagnosis in clinical scenarios where invasive fungemia is unexpected or isn’t contemplated in initial guidelines, such as in the described cases. Upon admission, patient 1 presented important risk factors such as *Candida* colonization, exposure to antibiotics and use of a double-J catheter. Patient 2, in turn, presented long hospital stay, previous antimicrobial therapy and an emergency gastrointestinal surgical procedure. Other potential risk factors include hemodialysis, necrotizing pancreatitis, central venous catheter, total parenteral nutrition, diabetes mellitus and immunodeficiency (neutropenia < 500 neutrophils/mL, hematologic malignancy, chemotherapy, immunosuppressant drugs) [19, 20]. A fundamental point, moreover, is not to underestimate the diagnostic value of cyst puncture/culture in difficult cases. In intra-abdominal candidiasis, only 4–20% of cases will be candidaemic [21]. To optimize blood culture sensitivity, 40–60-mL blood collection is recommended in adults.

*Candida* spp. grow very well in standard blood culture broths [22]. The detection of fungal antigen has been a useful approach to the presumptive diagnosis of invasive fungal infection in populations other than ADPKD. In patients with blood-culture-negative invasive candidiasis, mainly with intra-abdominal infection, the 1,3-β-D-glucan assay presented a sensitivity of 88% [23]. Some false-positive results, however, have been reported due to cross-reactions with certain hemodialysis filters, a caveat that could limit its application in patients undergoing hemodialysis support [24, 25].

Only less than 5% of *Candida albicans* isolates are resistant to azole [26]. This finding was corroborated by a retrospective study that analyzed the susceptibility of 153 *Candida albicans* isolates to three different antifungal drugs [27]. Only 1% of these isolates were resistant to fluconazole and 3% presented dose-dependent susceptibility; 96% of them, therefore, were susceptible to fluconazole. This same study showed that all isolates were susceptible to anidulafungin and only one of the 153 was resistant to amphotericin B. We chose, however, not to attempt these therapies in our first case due to the following reasons: 1) In a previously reported *C. kruzei* kidney cyst infection, none of the six analyzed cysts presented amphotericin B levels that reached the MIC level for the *C. krusei* recovered isolate [13]. The patient from this case report was, in fact, eventually submitted to bilateral nephrectomy; and 2) It is important to point out that none of the echinocandins are excreted in the urine as an active drug, therefore these drugs are not contemplated in *Candida* urinary tract infection treatment algorithms [28].

Notably, we now report two cases of fungal CI, a very unusual form of this complication. To our knowledge, these are the first two documented cases of *Candida albicans* renal CI; one with successful response to antifungal therapy and another with no improvement following this therapeutic approach, requiring nephrectomy to be resolved. Our report highlights fungi as potential etiologic agents for CI in ADPKD and the importance of including this possibility within the differential diagnosis in specific circumstances and clinical courses. Immunodeficient patients, previous prolonged antibiotic use and *Candida* colonization should call attention to this potential diagnosis. In addition, the identification of two cases of *Candida albicans* renal CI by our group raises the possibility that fungal CI may not be as rare as currently assumed. The relevance of such considerations is particularly high given that first and second-line antibiotic regimens currently applied to CI in ADPKD do not include antifungal agents. The efficacy of antifungal therapeutic strategies according to the identified specimen and its susceptibility and, most importantly, their corresponding intracystic antifungal levels, however, remain to be established.

## Data Availability

All meaningful data generated or analyzed in this study are included in the manuscript.

## Abbreviations

ADPKD: Autosomal Dominant Polycystic Kidney Disease
AmB: Amphotericin B
CI: Cyst Infection
CRP: C-reactive protein
CT: Computed Tomography
FDG: F-18 (^18^F) Fluor-deoxi-glucose
MRI: Magnetic Resonance Imaging
PET-CT: Positron Emission Tomography/Computed Tomography
UTI: Urinary Tract Infection
